# Use of a vibrating mesh nebulizer for allergen challenge

**DOI:** 10.1186/s13223-019-0392-8

**Published:** 2019-11-26

**Authors:** Donald W. Cockcroft, Beth E. Davis, Christianne M. Blais, Louis-Philippe Boulet, Marie-Éve Boulay, Hélène Villeneuve, Gail M. Gauvreau, Paul M. O’Byrne, Karen J. Howie, Caitlin D. Obminski

**Affiliations:** 1 Department of Medicine, University of Saskatchewan, Saskatoon, SK Canada; 2 Institut Universitaire de Cardiologie et de Pneumologie de Québec, Université Laval, Québec City, QC Canada; 3 Department of Medicine, McMaster University, Hamilton, ON Canada

**Keywords:** Allergen inhalation test, Methacholine inhalation test, Skin test endpoint, Jet nebulizer (Wright^®^), Vibrating mesh nebulizer (Solo^®^)

## Abstract

**Background:**

Allergen inhalation tests are a valuable research tool. The allergen dose producing an early asthmatic response (EAR) can be predicted from methacholine responsiveness and allergen skin test endpoint (STE). The Wright^®^ jet nebulizer, which is both inefficient and increasingly difficult to obtain, has been used historically. We assessed the Solo^®^ vibrating mesh nebulizer as an alternative for allergen and methacholine challenges.

**Methods:**

Eighteen mild atopic asthmatics completed the study. Doubling concentration allergen prick skin tests were performed to determine the STE in allergen units/mL. The Wright^®^ protocol was used to measure the methacholine provocation dose causing a 20% forced expired volume in one second (FEV_1_) fall (PD_20_) (μg) and the allergen PD_20_ (units). The Solo^®^ protocol (0.5 mL nebulized to completion, tidal breathing inhalation) was used to determine both methacholine PD_20_ and allergen PD_20_. The nebulizer order was randomized and separated by ≥ 2 weeks.

**Results:**

All data were log transformed. The allergen PD_20_, predicted from the methacholine PD_20_ and the STE, was within 2 doubling doses of the PD_20_ measured with the Wright^®^ and 2.64 doubling doses of that measured with Solo^®^. The Wright^®^ allergen PD_20_ correlated with the Wright^®^ methacholine PD_20_ (r = 0.74) and the STE (r = 0.78) and more strongly with the product of the two (Wright^®^ methacholine PD_20_ × STE, r = 0.91, p < 0.00001). The Solo^®^ allergen PD_20_ showed similar relationships with the Solo^®^ methacholine PD_20_ (r = 0.61), the STE (r = 0.75) and the product of the two (Solo^®^ methacholine PD_20_ × STE, r = 0.83, p < 0.00002). The Wright^®^ and the Solo^®^ methacholine geometric mean PD_20_s were not significantly different (49.3 and 54.5 μg respectively, p = 0.62). The Wright^®^ allergen PD_20_ was slightly but significantly lower than the Solo^®^ allergen PD_20_ (geometric means 6.7 and 10.5 units respectively, p = 0.003).

**Conclusion:**

The Solo^®^ allergen PD_20_ showed the same relationship with methacholine responsiveness and STE as did the Wright^®^. The Solo^®^ allergen PD_20_ was slightly but significantly higher than the Wright^®^ allergen PD_20_. The Solo^®^ vibrating mesh nebulizer was well tolerated and is an acceptable alternative for allergen challenge.

*Trial registration* clinicaltrials.gov: NCT03491358

## Background

Allergen inhalation challenge is a valuable research tool for the study of asthma pathophysiology and investigational new drug efficacy [1]. The early asthmatic response (EAR) to allergen depends on (non-allergic) airway responsiveness and the level of allergen-specific IgE [2]. It has been previously demonstrated that the concentration/dose of allergen required to produce a threshold EAR of a 20% decline in forced expired volume in one second (FEV_1_) can be predicted within 2–3 concentrations using the level of airway responsiveness measured by methacholine or histamine provocation and the level of allergen specific IgE assessed by the allergen skin test endpoint titration (STE) [3]. A caveat for this prediction is the requirement for methacholine and allergen to be inhaled in the same fashion using the same type of nebulizer, calibrated to the same weight loss.

Historically, the Wright^®^ jet nebulizer (Roxon Medi-Tech, St. Leonard, QC) calibrated to run at a weight loss of 0.13 g/min with inhalation performed by two minutes of tidal breathing [4] has been used for both methacholine and allergen inhalation. The Wright^®^ nebulizer is inefficient (approximately 75% of weight loss is evaporation [5, 6]), expensive, non-disposable, and increasingly difficult to acquire. The Aerogen Solo^®^ vibrating mesh nebulizer, referred to as the Solo^®^ throughout, (Aerogen Ltd, Galway Ireland) features no evaporation and has been validated for use in methacholine challenge testing [7, 8]. The current study was designed to assess the Solo^®^ vibrating mesh nebulizer for use in the standardized allergen challenge protocol performed in AllerGen National Centres of Excellence (NCE) Clinical Investigator Collaborative (CIC) studies and to compare it to the current Wright^®^ jet nebulizer protocol.

## Methods

### Participants

Eligible participants had mild atopic asthma requiring only infrequent inhaled β_2_ agonist, an FEV_1_ > 70% predicted, and a methacholine provocation dose causing a 20% fall in FEV_1_ (PD_20_) ≤ 400 μg. Participants were non-smokers with < 10 pack year cumulative smoking history. Individuals who were pregnant, lactating, who had relevant allergen exposure or respiratory tract infection within the previous 4 weeks or who had significant medical conditions were excluded. Ethics approval was received from each study site and signed informed consent was obtained prior to study entry.

### Study design

Participants attended the laboratory on 5 occasions. Visit 1 visit was to assess eligibility, to obtain signed consent, to perform baseline spirometry and screening allergen skin prick tests and from these select the best allergen for inhalation testing. The selected allergen was one which was clinically relevant to the participant and which produced a large (≥ 5 mm) wheal. The STE for the selected allergen was measured at Visit 2 to allow prediction of the starting allergen concentration for inhalation in conjunction with the methacholine response [3]. At Visits 2 and 3 the methacholine PD_20_ and the allergen PD_20_ were measured respectively both with either the Wright^®^ or the Solo^®^ nebulizer. After a minimum 2-week washout, at Visits 4 and 5 these challenges were repeated with the other nebulizer. The order of the nebulizers was randomized.

### Skin test endpoint (STE) titration

The STE was determined as previously outlined [9]. Allergens (Omega Laboratories, Montreal QC) were dispensed in protein nitrogen units/mL, biologic allergen units/mL or allergen units/mL. For conformity the allergen dose was expressed in “units”. Allergens were diluted two-fold from 1:8 to 1:1024 or beyond if required and the dilutions were labeled as the allergen concentration in units/mL. Duplicate skin prick tests were performed, the mean wheal diameter measured at 10 min, and the STE recorded as the weakest concentration (units/mL) causing a 2 mm mean wheal diameter.

### Methacholine inhalation tests

The standard Wright^®^ nebulizer methacholine test [4, 10] was done as follows. The nebulizer was calibrated to a weight loss of 0.13 g/min. Complete spirometry was initially measured in triplicate. Isotonic saline was then inhaled by tidal breathing for 2 min, and the FEV_1_ (truncated manoeuvre to avoid fatigue) measured at 30 and 90 s. Doubling concentrations of methacholine were then inhaled in the same manner at 5 min intervals with FEV_1_ repeated at 30 and 90 s The available concentrations ranged from 0.031 to 64 mg/mL; the starting concentration for an individual was selected based on previous testing if available or based on validated guidelines [10, 11]. Inhalations were stopped when the FEV_1_ had fallen ≥ 17% and the provocation concentration causing a 20% FEV_1_ fall (PC_20_) was interpolated from the last 2 data points [10] or extrapolated from the last data point [12]. The PC_20_ was converted to a PD_20_ (μg) based on several studies documenting that a Wright^®^ PC_20_ of 16 mg/mL equates to a PD_20_ of 400 μg [6–8, 13, 14]. The accepted values for a normal (negative) methacholine challenge test are PC_20_ and PD_20_ > 16 mg/mL and > 400 μg respectively [15].

The Solo^®^ methacholine challenge was done as previously described [8]. Doubling doses of methacholine were delivered by nebulizing 0.5 mL of methacholine to completion and inhaling by tidal breathing; this requires 90 to 180 s [8]. A concentration of 2 mg/mL × 0.5 mL × 0.4 (respiratory duty cycle [16]) exposes the individual to 400 μg. Following saline inhalation appropriate doubling concentrations up to 4 mg/mL (= 800 μg) were used. The remainder of the challenge (timing of FEV_1_ measurements, time between doses, calculation of the PD_20_, etc.) was identical to the Wright^®^ method.

### Allergen inhalation tests

Allergen inhalation tests were done as previously described using the Wright^®^ nebulizer [17]. Spirometry was measured in triplicate. Doubling concentrations of allergen were then inhaled (2 min of tidal breathing, nebulizer calibrated to a weight loss of 0.13 g/min and starting 3 or 4 concentrations below the predicted EAR concentration [3]) at 12 min intervals until the FEV_1_ measured at 10 min after inhalation had fallen ≥ 15%. At an FEV_1_ fall between 15 and 20% the FEV_1_ was repeated 10 min later before giving another concentration if required. The allergen PC_20_ (units/mL) was converted to allergen PD_20_ (units) assuming a similar relationship as seen with methacholine. After PD_20_ measurement, participants received a single inhaled dose of salbutamol 200 μg to reverse bronchoconstriction and a single inhaled dose of fluticasone propionate 500 μg to prevent development of the late asthmatic response [18].

The Solo^®^ allergen challenge was performed in an analogous manner. Assuming a similar relationship for dose comparison between the Solo^®^ and the Wright^®^ nebulizers seen with methacholine, the starting allergen concentration was 3 doubling concentrations below the starting concentration used for the Wright^®^ (i.e. 6–7 concentrations below the Wright^®^ prediction). The allergen, 0.5 mL, was nebulized to completion and inhaled by tidal breathing; the remainder of the challenge protocol was identical to the Wright^®^ protocol; the result was expressed as the allergen PD_20_ in units.

### Analysis

Statistics were done using a computerized statistics programme (Statistix 9 (Analytical Software, Tallahassee, FL, USA). PD_20_ and STE values were log transformed prior to analysis. The Student’s paired t test was used for comparison of means. Linear regression analysis was used for the following (all values logged):

- Measured Wright^®^ allergen PD_20_ vs Predicted allergen PD_20._
- Wright^®^ allergen PD_20_ vs Wright^®^ methacholine PD_20._
- Wright^®^ allergen PD_20_ vs STE.
- Wright^®^ allergen PD_20_ vs (Wright^®^ methacholine PD_20_ × STE).
- Measured Solo^®^ allergen PD_20_ vs Predicted allergen PD_20._
- Solo^®^ allergen PD_20_ vs Solo^®^ methacholine PD_20._
- Solo^®^ allergen PD_20_ vs STE.
- Solo^®^ allergen PD_20_ vs (Solo^®^ methacholine PD_20_ × STE).

## Results

Eighteen participants, all poylsensitized but with no current allergen exposure (except house dust mite), completed the study without adverse events. Three additional enrolled participants did not complete the study; one because the FEV_1_ was < 70% at Visit 1, one because the methacholine PD_20_ was > 400 μg at Visit 2, and one because of a failure to respond to allergen (1:32 with the Solo^®^ equating to ~ 1:4 with the Wright^®^) at Visit 3. Anthropometric data, baseline FEV_1_, baseline methacholine PD_20_ and allergen used for challenges are shown in Table 1.

**Table 1:** Demographics, FEV_1_, methacholine PD_20_, and allergen used for inhalation

Participant	Site^a^	Sex	Age (year)	Height (cm)	Weight (kg)	FEV_1_ (L)	FEV_1_ (%)	Methacholine PD20 (μg)	Allergen
1	S	M	42	178	86.4	3.50	84	29.1	Cat
2	S	M	24	170	81.8	4.15	98	82.7	Cat
3	S	F	25	170	70.9	3.53	98	55.4	Cat
4	S	M	27	170	79.5	3.60	87	81.5	Cat
5	S	F	23	162	48.6	2.68	82	118	Mite
6	S	F	42	158	54.5	2.49	90	10.5	Mite
7	L	M	42	173	67.9	3.44	88	171	Birch
8	L	F	30	162	66.9	3.66	115	239	Cat
9	L	M	35	182	63.1	3.78	82	142	Cat
10	L	F	38	157	77.6	2.61	92	8.9	Cat
11	L	F	25	152	83.0	3.16	112	1.9	Cat
12	L	M	34	186	73.2	3.66	88	26.6	Horse
13	M	M	58	184	102.0	3.69	92	162	Mite
14	M	M	54	175	76.0	3.40	95	80.6	Cat
15	M	M	28	181	87.9	4.57	98	18.1	Grass
16	M	F	21	174	72.5	3.08	82	46.5	Grass
17	M	F	25	161	69.5	2.50	78	265	Ragweed
18	M	F	30	167	80.0	3.36	101	29.4	Mite
Mean			33.5	170.1	74.5	3.38	92.3	49.3^b^	
SD			10.7	10.0	12.4	0.55	10.1	(25.8–94.0)	

^a^Site: S = Saskatchewan, L = Laval, M = McMaster

^b^Geometric mean (95% confidence intervals)

### Wright^®^

**Fig. 1:**
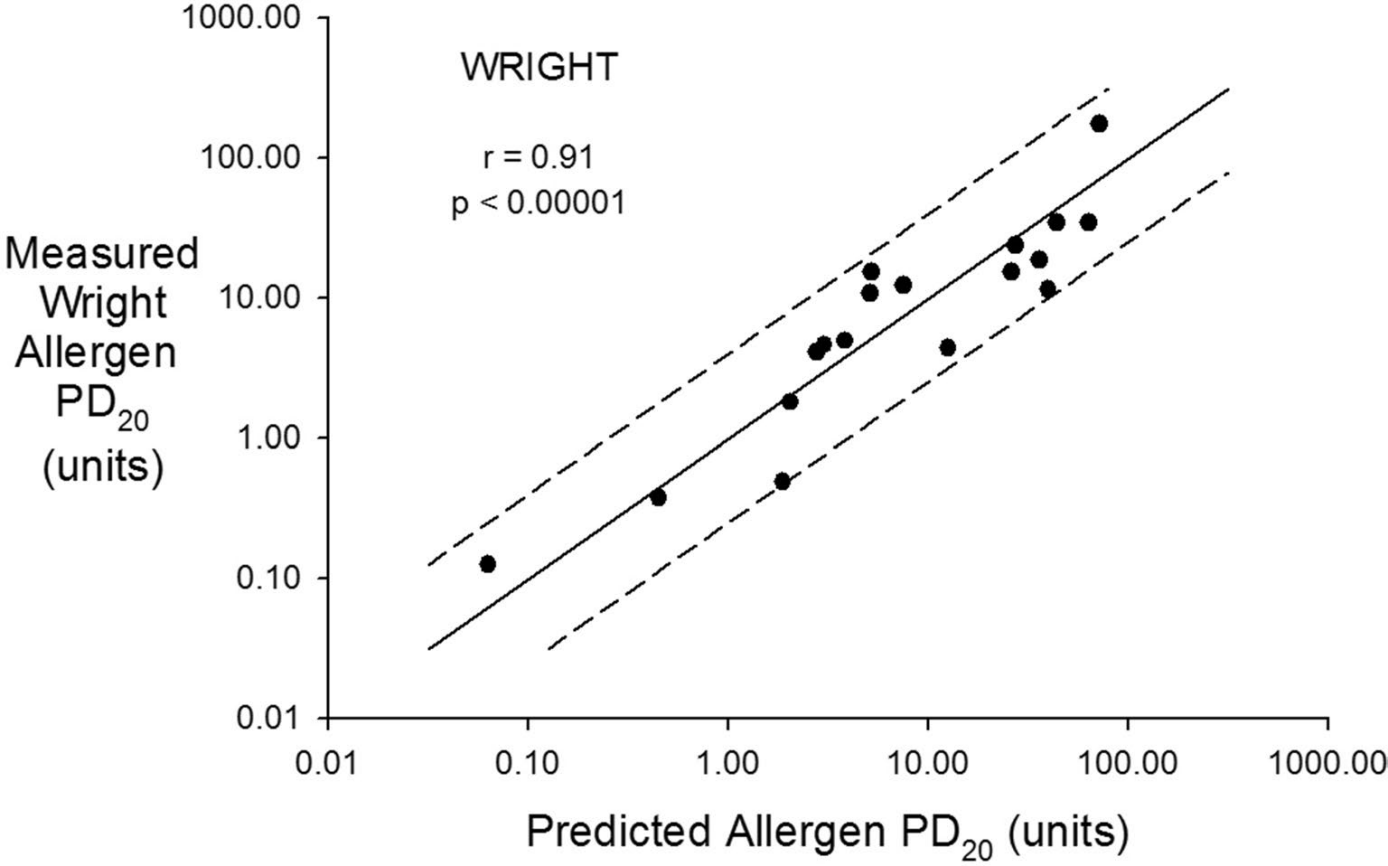
Measured Wright^®^ allergen PD_20_ (units) on the vertical axis and predicted allergen PD_20_ (units) on the horizontal axis both plotted in a log scale. The solid line is the line of identity and the dashed lines represent ± 2 doubling doses

**Fig. 2:**
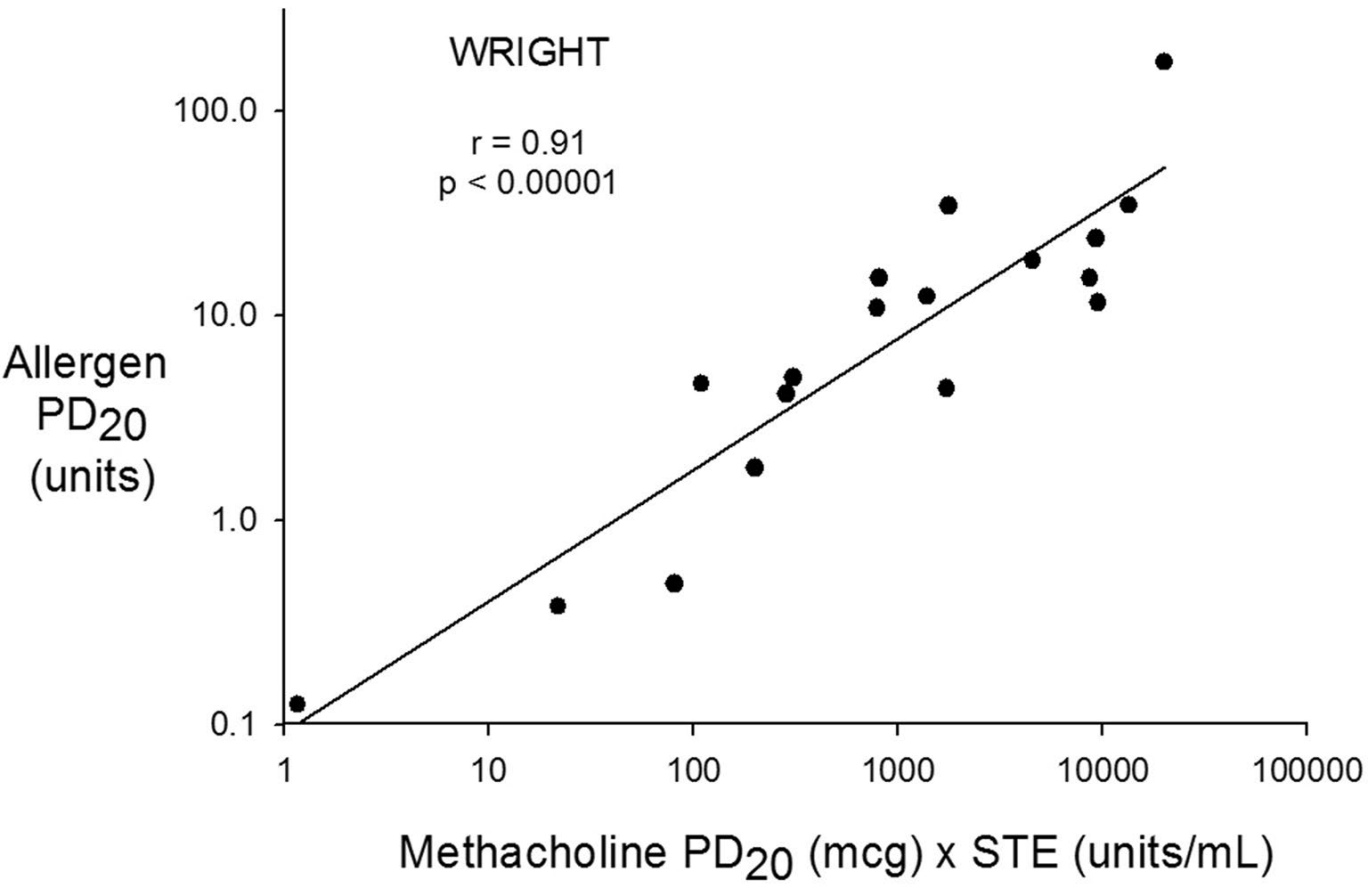
Wright^®^ allergen PD_20_ on the vertical axis and Wright^®^ Methacholine PD_20_ × STE on the horizontal axis both plotted on a log scale. The regression equation is; Log Allergen PD_20_ (units) = − 1.03 + 0.64 × log (Methacholine PD_20_ [μg] × STE [units/mL])

The measured allergen PD_20_ correlated with the predicted allergen PD_20_ (r = 0.91, p < 0.00001) and all predictions were within 2 (maximum 1.96) doubling doses of the measured allergen PD_20_ (Fig. 1). The geometric means for the measured and predicted PD_20_ values were 6.7 units (95% CI 2.7–15.8) and 7.0 units (95% CI 2.5–17.7) respectively (p = 0.68). The allergen PD_20_ (units) correlated with both the methacholine PD_20_ (r = 0.74) and the STE (r = 0.78,). Allergen PD_20_ correlated more strongly with the product of the methacholine PD_20_ (μg) and the STE (units/mL) (r = 0.91, p < 0.00001) (Fig. 2).

### Solo^®^

**Fig. 3:**
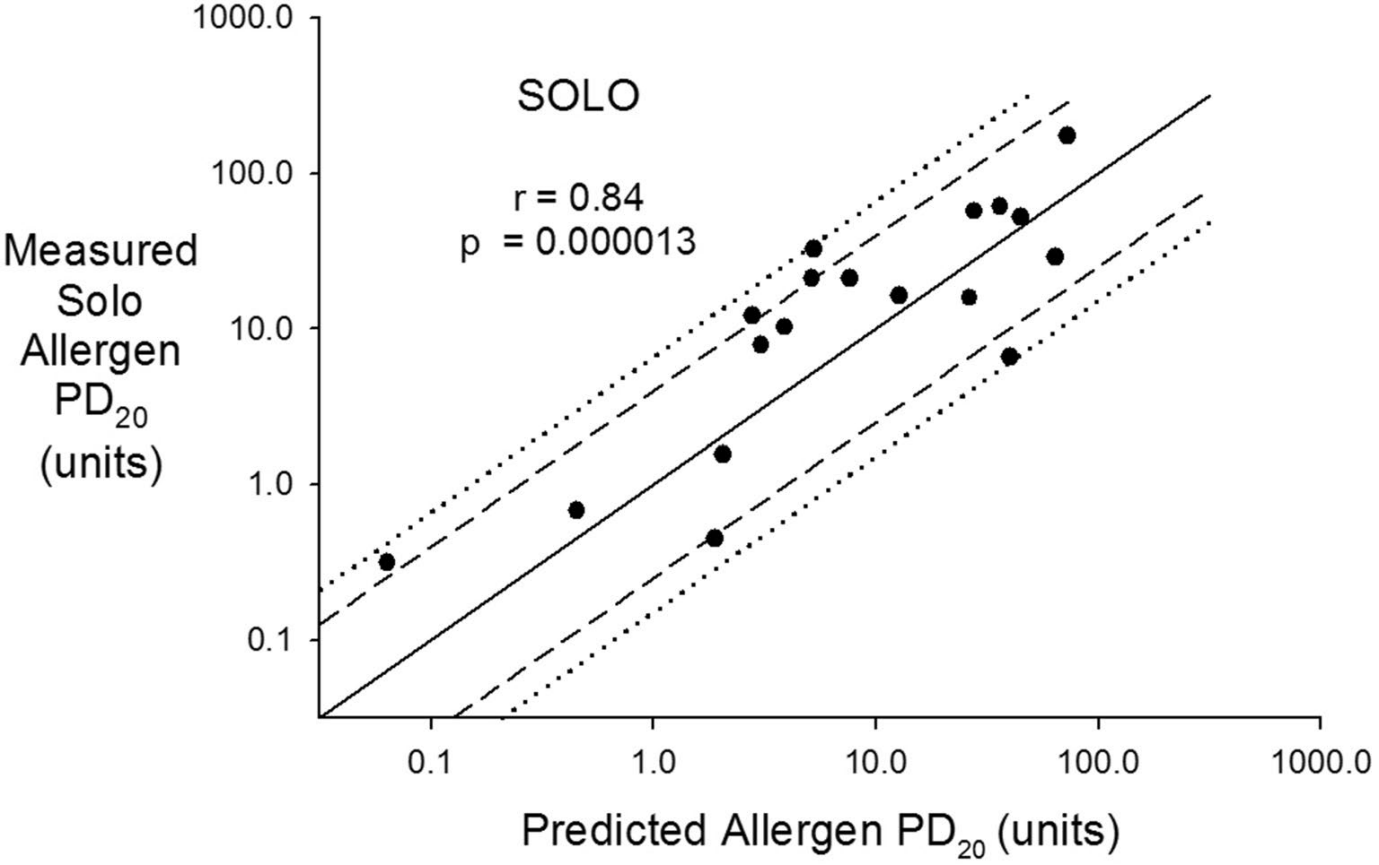
Measured Solo^®^ allergen PD_20_ (units) on the vertical axis and predicted allergen PD_20_ (units) on the horizontal axis both plotted in a log scale. The solid line is the line of identity, the dashed lines represent ± 2 doubling doses and the dotted lines ± 2.64 doubling doses

**Fig. 4:**
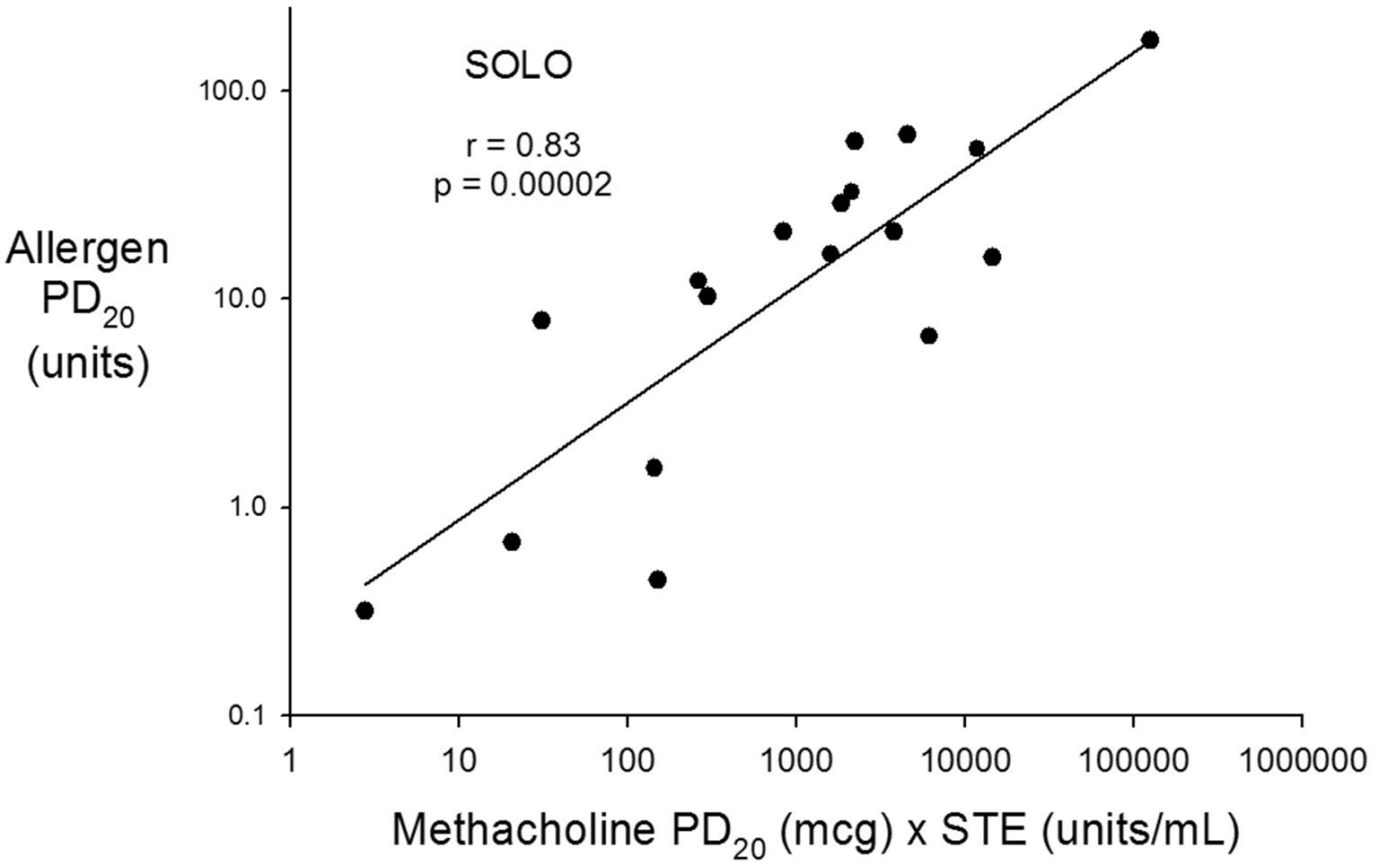
Solo^®^ allergen PD_20_ on the vertical axis and Solo^®^ Methacholine PD_20_ × STE on the horizontal axis plotted on a log scale. The regression equation is; Log Allergen PD_20_ (units) = − 0.62 + 0.56 × log (Methacholine PD_20_ [μg] × STE [units/mL])

The measured Solo^®^ allergen PD_20_ correlated with the predicted allergen PD_20_ (r = 0.84, p = 0.000013) and was within 2 doubling does of the predicted allergen PD_20_ in 14 of 18 and within 2.64 doubling doses in all 18 (Fig. 3). Similar to the Wright^®^, the Solo^®^ allergen PD_20_ correlated with both the Solo^®^ methacholine PD_20_ (r = 0.61) and the STE (r = 0.75) and more strongly with the product of the 2 (r = 0.83, p = 0.00002) (Fig. 4).

### Wright^®^ Solo^®^ comparisons

**Fig. 5:**
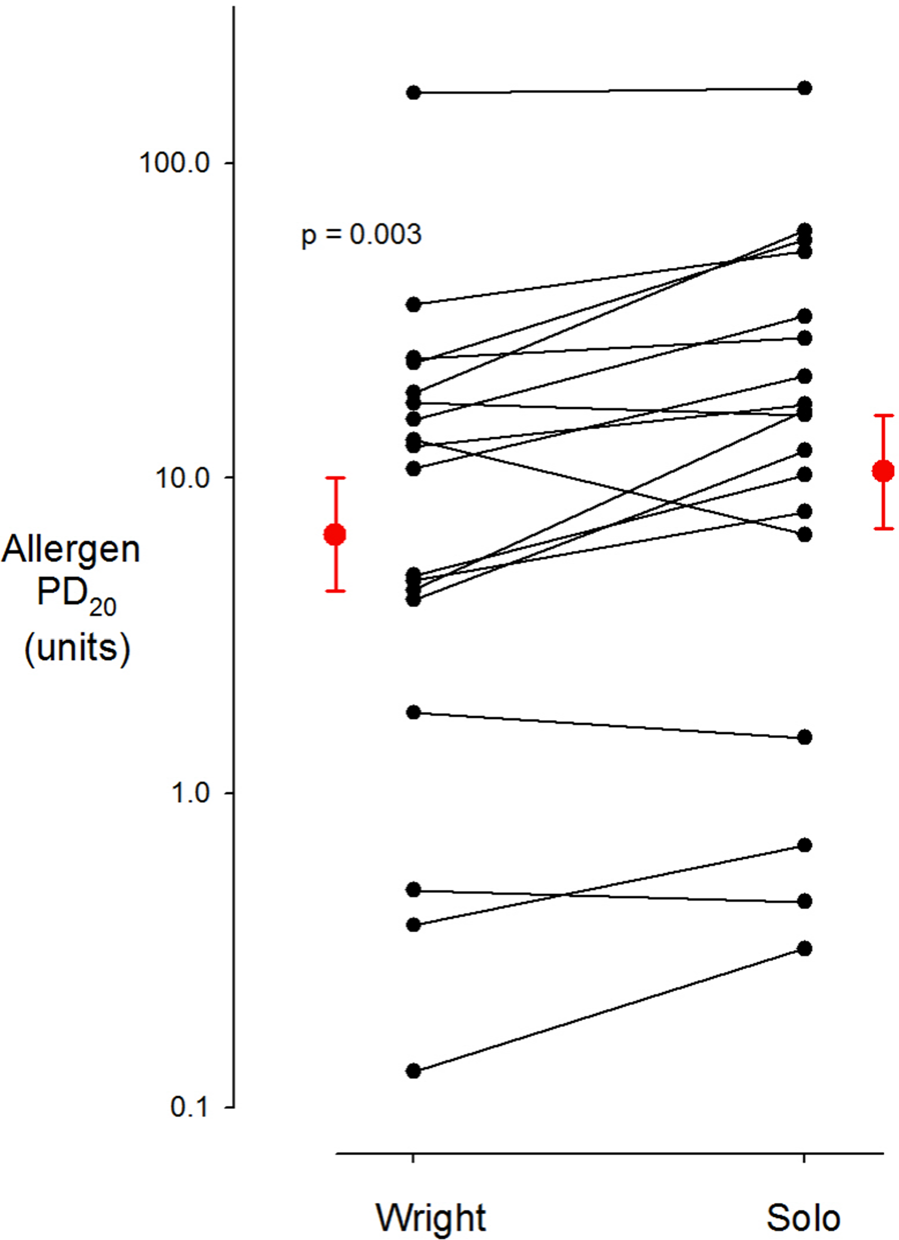
Individual values for Wright^®^ allergen PD_20_ on the left and Solo^®^ allergen PD_20_ on the right. The red points are the geometric means with standard error bars. The Wright^®^ allergen PD_20_ is slightly but significantly smaller than the Solo^®^ allergen PD_20_ (6.7 vs 10.5 units respectively, p = 0.003)

The Wright^®^ and the Solo^®^ methacholine PD_20_s were not significantly different with geometric means of 49.3 (95% CI 25.8–94.0) and 54.2 μg (95% CI 26.7–110) respectively (p = 0.62). The geometric mean Wright^®^ allergen PD_20_, 6.7 units (95% CI 2.7–15.8), was slightly but significantly lower than geometric mean Solo^®^ allergen PD_20_, 10.5 units (95% CI 4.4–25.1), (p = 0.003). Individual values for the Wright^®^ and the Solo^®^ allergen PD_20_s are shown in Fig. 5. There was no sequence effect (i.e. nebulizer order) nor was there any difference between the three sites.

## Discussion

These data indicate that the Solo^®^ vibrating mesh nebulizer can be successfully used for performance of allergen inhalation tests. Allergen responsiveness showed the same relationship with methacholine responsiveness and level of allergen sensitivity as was seen with the Wright^®^ nebulizer protocol. The measured Solo^®^ allergen PD_20_ was within 2.64 doubling doses of the prediction.

In 1987, an equation was developed to predict the dilution/concentration of allergen that would produce a 20% EAR [3]. This was based on the histamine PC_20_ (mg/mL) and the allergen skin test endpoint (dilution) producing a 2 mm wheal. Both allergen and histamine were inhaled with 2 min of tidal breathing from a Wright^®^ nebulizer calibrated in the same manner. Methacholine was subsequently substituted for histamine in the same concentration since histamine and methacholine PC_20_s are identical in asthmatics [19]. In the original study, the equation successfully estimated the dilution required for an EAR within 2 doubling dilutions in 92% and 3 doubling dilutions in 100% of challenges [3]. The equation and a sample calculation are below:

$${\text{Log}}\;{\text{predicted}}\;{\text{allergen}}\;{\text{dilution}}\, = \,0.68\log \left( {{\text{methacholine}}\;{\text{PC}}_{20} \, \times \,{\text{STE}}\;{\text{dilution}}} \right)$$

For example with a methacholine PC_20_ of 2.2 mg/mL and an STE of 1/1024.

$${\text{Log}}\;{\text{predicted}}\;{\text{allergen}}\;{\text{dilution}}\, = \,0.68\log \left( {2.2\, \times \,1/1024} \right)\, = \, - 1.81$$

$${\text{Predicted}}\;{\text{allergen}}\;{\text{dilution}}\, = \,{\text{antilog}}\left( { - 1.81} \right)\, = \,0.0155\,\sim \,1/64\;{\text{dilution}}.$$

This prediction is routinely used as a guide for allergen challenge tests. A starting allergen dilution of 3 or occasionally 4 doubling dilutions below the prediction has proved a safe and effective method for allergen challenges performed in AllerGen NCE CIC and other studies. The purpose is to allow some test shortening when compared to methods advocating starting with the weakest allergen dilution causing a 2 mm wheal skin test response [20]. The current study validates this prediction equation when applied to the Wright^®^ data, since all measured values were within 2 doubling doses of the prediction. Despite the slightly higher measured Solo^®^ allergen PD_20_ vs the Wright^®^ (i.e. slightly *less* responsive), 78% of values were within 2 doubling doses and 100% within 2.64 doubling doses. This would suggest that the 1987 prediction equation can be safely and effectively used (with modification for the units and nebulizer differences) until such time as there are enough data to develop a “Solo^®^ specific” equation.

The major strength of this study is the experienced group of investigators at the three sites. The one weakness is the inability to assess the solute output of the jet (Wright®) nebulizer. Based on the known evaporative features [5, 6], and both breath simulation testing [6] and clinical challenge testing [7, 8, 12, 13] it is reasonable to equate a methacholine PC_20_ of 16 mg/mL to a methacholine PD_20_ of 400 μg. The current study validates this, since using this conversion the Wright^®^ and Solo^®^ methacholine PD_20_s were essentially identical. However, data are currently lacking for nebulized allergen and it is possible that allergen solutions could be handled differently by the nebulizers.

## Conclusion

In summary the Solo^®^ vibrating mesh nebulizer, proved to be a safe, effective and well tolerated device for administering inhaled allergen. This provides a valuable alternative to the Wright^®^ jet nebulizer.

## Data Availability

All data are available from the corresponding author on reasonable request don.cockcroft@usask.ca.

## Abbreviations

STE: skin (prick) test endpoint
FEV_1_: forced expired volume in one second
PC_20_: provocation concentration causing a 20% FEV_1_ fall
PD_20_: provocation dose causing a 20% FEV_1_ fall
EAR: early asthmatic response
SD: standard deviation
CI: confidence interval
NCE: National Centres of Excellence
CIC: Clinical Investigator Collaborative
